# Characteristics and outcomes of older HIV-infected patients receiving antiretroviral therapy in Malawi: A retrospective observation cohort study

**DOI:** 10.1371/journal.pone.0180232

**Published:** 2017-07-07

**Authors:** Hannock Tweya, Caryl Feldacker, Tom Heller, Salem Gugsa, Wingston Ng’ambi, Omisher Nthala, Mike Kalulu, Jane Chiwoko, Rabecca Banda, Agness Makwinja, Sam Phiri

**Affiliations:** 1 The International Union Against Tuberculosis and Lung Disease, Paris, France; 2 Lighthouse Trust, Lilongwe, Malawi; 3 International Training and Education Center for Health, University of Washington, Seattle, Washington, United States of America; 4 Department of Medicine, University of North Carolina School of Medicine, Chapel Hill, North Carolina, United States of America; National and Kapodistrian University of Athens, GREECE

## Abstract

**Objective:**

To estimate patients enrolling on antiretroviral therapy (ART) over time; describe trends in baseline characteristics; and compare immunological response, loss to follow-up (LTFU), and mortality by three age groups (25–39, 40–49 and ≥50 years).

**Design:**

A retrospective observation cohort study.

**Methods:**

This study used routine ART data from two public clinics in Lilongwe, Malawi. All HIV-infected individuals, except pregnant or breastfeeding women, aged ≥ 25 years at ART initiation between 2006 and 2015 were included. Poisson regression models estimated risk of mortality, stratified by age groups.

**Results:**

Of 37,378 ART patients, 3,406 were ≥ 50 years old. Patients aged ≥ 50 years initiated ART with more advanced WHO clinical stage and lower CD4 cell count than their younger counterparts. Older patients had a significantly slower immunological response to ART in the first 18 months on ART compared to patients aged 25–39 years (p = 0.04). Overall mortality rates were 2.3 (95% confidence Interval (CI) 2.2–2.4), 2.9 (95% CI 2.7–3.2) and 4.6 (95% CI 4.2–5.1) per 100 person-years in patients aged 25–39 years, 40–49 years and 50 years and older, respectively. Overall LTFU rates were 6.3 (95% CI 6.1–6.5), 4.5 (95% CI 4.2–4.7), and 5.6 (95% CI 5.1–6.1) per 100 person years among increasing age cohorts. The proportion of patients aged ≥ 50 years and newly enrolling into ART care remained stable at 9% while the proportion of active ART patients aged ≥50 years increased from 10% in 2006 to 15% in 2015.

**Conclusion:**

Older people had slower immunological response and higher mortality. Malawi appears to be undergoing a demographic shift in people living with HIV. Increased consideration of long-term ART-related problems, drug-drug interactions and age-related non-communicable diseases is warranted.

## Introduction

As scale-up of antiretroviral therapy (ART) continues in sub-Saharan Africa (SSA), increasing numbers of older people will start ART and need long-term ART care. By 2040, the number of HIV-infected people aged 50 years or older will nearly triple in SSA from an estimated 3.1 million in 2011 to 9.1 million. These older people will account for 25% of all infections in the region [1].

Previous research in developed countries suggests that older HIV-infected people receiving ART have different characteristics at diagnosis and clinical outcomes compared to younger HIV-infected people. Older HIV-infected people have more advanced AIDS-related conditions at diagnosis [2,3], faster HIV progression to AIDS and higher mortality than their young counterparts [4,5]. In addition, older HIV-infected individuals mount slower immunological response to ART and have more frequent ART-related toxicities[6,7]. Despite these findings suggesting the existence of age-related disparities, other studies contradicted these, noting similar findings of all ages [8–10].

In sub-Saharan Africa, there is a paucity of research on older populations on ART [11,12]. Among the few studies that described baseline characteristics, immunological response and mortality among older patients starting ART, small sample sizes [13,14] and high loss to follow-up (LTFU) [15,16] limit generalizability of the findings. Furthermore, few studies empirically explored the increase in numbers of older HIV-infected cohorts receiving ART in resource-limited settings[15][17]. The evidence base, overall, is sparse and inconclusive. We, therefore, (i) estimated the proportions of HIV-infected patients newly enrolling and active on ART by three age groups (25–39, 40–49 and ≥50 years) over time, (ii) described trends in baseline characteristics and (iii) compared immunological responses, LTFU and mortality across the age groups from a large, ART cohort in Lilongwe, Malawi.

## Methods

### Study design, population and setting

This retrospective cohort study uses data from routine ART services delivered at Lighthouse Trust’s two public HIV clinics, Lighthouse and Martin Preuss Centre (MPC). Lighthouse and MPC are described in detail elsewhere [18]. In brief, the clinics are high-patient load facilities located in Malawi’s capital, Lilongwe. Both clinics use a real-time, point-of-care, electronic medical record system (EMRs) for patient management where the study data were obtained. Combined, Lighthouse and MPC have 31,311 ART patients, respectively.

All HIV-infected individuals, aged 25 years and older, who initiated ART between January 2006 and December 2015 were included in the study. Pregnant or breastfeeding women were excluded as criteria for ART initiation differed from the other HIV-infected individuals.

### Data collection through the EMRs

During the study period, all individuals diagnosed with HIV were registered for ART care in the EMRs. Patients were initiated on ART based on WHO clinical staging (3 or 4) or CD4 cell count if they had WHO clinical stage 1 or 2 conditions [19–21]. ART eligibility based on CD4 cell count changed during the study period: CD4 cell counts ≤ 200 cells/μl was used before 2008, CD4 cell counts ≤ 250 cells/μl between 2008 and 2010, CD4 cell counts ≤350 cells/μl between 2011 and 2013 and CD4 cell counts ≤ 500 cells/μl in 2014 and 2015. Between January 2006 and June 2011, all ART eligible patients were initiated on Stavudine Lamivudine and Nevirapine and from July 2011 onwards on Tenofovir Lamivudine and Efavirenz, as first line regimen. Routine return visits were scheduled monthly during the first six months on ART and every two or three months thereafter if no clinical complications occurred. CD4 cell count monitoring was used between 2006 and 2013, thereafter, virological monitoring was used. CD4 cell counts were performed every 6 months after ART initiation. Some patients had no CD4 counts at specific monitoring milestones due to the unavailability of CD4 count testing services. Deaths were generally updated retrospectively after active tracing of patients who missed their appointments. Patients were classified as LTFU if they did not return to the clinic after 60 days from the day they were expected to run out of ARVs.

### Statistical analysis

Patients were categorised into three age groups: 25–39, 40–49 and ≥50 years. Trends in baseline characteristics, mortality and LTFU were compared by age at ART initiation. Age at the beginning of the calendar year was used in determining proportions of active HIV-infected patients on ART each year. For person-time calculation, the analysis period began when patients initiated on ART at the facility. Patients who transferred in on ART from another facility were considered to come under observation when first registered at the study clinics, and their previous time on ART was accounted for. Observation of ‘time at risk’ of mortality or LTFU ended either at the time of death, LTFU or the censoring date (31^st^ December 2015). Poisson regression models were used to assess crude and adjusted associations between patients’ characteristics and mortality. To compare risk factors for death, we stratified the models by two age groups at ART initiation: 25–39 and ≥50 years. Gender was included in the multivariable models *a priori*. Significant factors at p <0.05 level in univariable models were included in multivariable models. Analysis was conducted using Stata 12.0.

### Ethical approval

The study was approved by the National Health Science Research Committee in Lilongwe, Malawi and the Ethics Advisory Group of the International Union Against Tuberculosis and Lung Disease in Paris, France. The ethics committees waived the need for patient consent because the study used routine program data and did not include any personal identifiers.

## Results

Between January 1, 2006 and December 31, 2015, 41,603 adult HIV-infected patients enrolled on ART at Lighthouse and MPC clinics, combined. Of these, 3,572 women were excluded because they started lifelong ART for PMTCT and 653 patients had no ARV dispensing visit after registering as transfer-in, leaving 37,378 patients eligible for this analysis.

Total follow-up time was 103,559 person-years with a median of 1.83 (Interquartile range (IQR) 0.50–4.49) years. A total of 3,406 patients (9%) initiated ART at age ≥ 50 years (Table 1). Overall, the cohort was 52% female, the proportion of females initiating ART decreased with age, with females comprising 41% of those aged ≥ 50 years. Median age at ART initiation was 35 years old (IQR 30–41). Median CD4 cell count at ART initiation was similar in patients aged 40–49 years (163 cells/μl) and ≥50 years (165 cells/μl) (*p* = 0.16) but lower compared to patients aged 25–39 years (174 cells/μl) (*p*<0.001). More older patients had WHO clinical stage 3 or 4 conditions than those aged 25–39 years at ART initiation (p <0.001). Proportions of patients with body mass index (BMI) < 16.0 and tuberculosis (TB) at ART initiation were similar across the age groups.

**Table 1:** Baseline characteristics, loss to follow-up and mortality, by age at ART initiation between 2006 and 2015 at Lighthouse and Martin Preuss clinics, Malawi.

Characteristics	Total		Age in years					
			25–39		40–49		≥50	
Patients enrolled	37,378	100%	26,155	70%	7,817	21%	3,406	9%
Women	19,600	52%	14,799	57%	3,390	43%	1,411	41%
Median CD4 count (IQR)^€^	174		178		163		165	
	(87–264)		(90–270)		(78–253)		(86–257)	
CD4 count categories								
0–199	12,192	57%	8,259	56%	2,778	60%	1,155	59%
200–349	6,688	31%	4,756	32%	1,337	29%	595	30%
350–499	1,887	9%	1,363	9%	363	8%	161	8%
≥500	507	2%	338	2%	120	3%	49	3%
WHO HIV clinical stage								
1 or 2	14,433	39%	10,455	41%	2,779	36%	1,199	35%
3	17,409	47%	11,849	46%	3,830	50%	1,730	52%
4	4,978	14%	3,458	13%	1,090	14%	430	13%
Tuberculosis								
TB at initiation	3,818	14%	2,677	14%	808	14%	333	14%
TB in the past 2 year	1,045	4%	683	4%	249	4%	113	5%
None	22,208	82%	15,654	82%	4,576	82%	1,978	81%
Missing	10,307	28%	7,141	27%	2,184	28%	982	29%
Kaposi’s Sarcoma								
Yes	1,103	3%	834	3%	208	3%	61	2%
No	36,275	97%	25,321	97%	7,609	97%	3,345	98%
Body mass index								
< 18.5	5,278	17%	3,747	17%	1,070	16%	461	16%
18.5–24.9	20,978	66%	14,860	67%	4,292	64%	1,826	63%
≥25.0	5,409	17%	3,484	16%	1,311	20%	614	21%
Missing	5,713	15%	4,064	16%	1,144	15%	505	15%
Year of ART initiation								
2006	2,216	6%	1,438	6%	559	7%	219	6%
2007	3,615	10%	2,499	10%	777	10%	339	10%
2008	3,954	11%	2,741	10%	836	11%	377	11%
2009	4,050	11%	2,863	11%	827	11%	360	11%
2010	4,334	12%	3,106	12%	857	11%	371	11%
2011	4,567	12%	3,271	13%	875	11%	421	12%
2012	3,930	11%	2,757	11%	811	10%	362	11%
2013	3,678	10%	2,576	10%	758	10%	344	10%
2014	3,762	10%	2,642	10%	791	10%	329	10%
2015	3,272	9%	2,262	9%	726	9%	284	8%
Outcome at closure of analysis								
Deaths	2,741	7%	1,663	6%	671	9%	407	12%
LTFU	6,062	16%	4,555	17%	1,014	13%	493	14%
Transfer-out	7,972	21%	5,592	21%	98	1%	801	24%
Stop ART	659	2%	524	2%	98	1%	37	1%
Alive on ART	19,944	53%	13,821	53%	4,555	57%	1,668	49%
Mortality per 100 person-years (95% CI)^β^	2.6		2.3		3.0		4.6	
	(2.5–2.7)		(2.2–2.4)		(2.7–3.2)		(4.2–5.1)	
Loss to follow-up (95% CI)	5.9		6.3		4.5		5.6	
	(5.7–6.0)		(6.1–6.5)		(4.2–4.7)		(5.1–6.1)	
Median follow-up time (years)	1.83		1.82		2.00		1.55	

ART: antiretroviral therapy; CI: confidence interval;

^€^CD4 tests were regularly done in patients not eligible based on WHO HIV clinical stage;

^β^ p < 0.001 for ages 25–39 vs 40–49 and ages 40–49 vs 50 and above;

Proportion of older HIV-infected patients initiating ART with WHO clinical stage 3 or 4, TB and KS decreased in each successive year between 2006 and 2015 (p < 0.01 for trend) (Table 2). We noted a similar decline in younger age groups (S1 Table). The proportion of older male patients initiating ART increased in each successive year during the study period, although this trend was not significant (p = 0.146 for trend).

**Table 2:** Baseline characteristics of adult patients (aged ≥50) starting ART, by year of ART initiation.

Baseline characteristics	Total (n (%))		Year of ART initiation										
			2006–2007		2008–2009		2010–2011		2012–2013		2014–2015		p-value*
Total enrolled	3406	9%	558	10%	737	9%	792	9%	706	9%	613	9%	
Male sex	1995	59%	313	56%	428	58%	473	60%	406	58%	375	61%	0.146
WHO HIV clinical stage													<0.001
1 or 2	1199	36%	84	15%	206	28%	252	32%	308	44%	349	59%	
3	1730	52%	381	69%	415	57%	423	54%	300	43%	211	35%	
4	430	13%	86	16%	108	15%	111	14%	89	13%	36	6%	
Median CD4 count (IQR)^€^	165 (86–257)		145 (77–224)		143 (74–220)		139 (70–233)		201(101–282)		260 (131–364)		
CD4 count													<0.001
0–199	1155	59%	247	68%	324	69%	276	66%	167	50%	141	38%	
≥200	805	41%	117	32%	147	31%	145	34%	167	50%	229	62%	
Missing	1446	42%	194	35%	266	36%	371	47%	372	53%	243	40%	
Body mass index													0.314
< 18.5	461	16%	72	16%	95	16%	130	19%	84	15%	80	14%	
≥18.5	2440	84%	391	84%	495	84%	572	81%	491	85%	491	86%	
Missing	505	15%	95	17%	147	20%	90	11%	131	19%	42	7%	
Tuberculosis													<0.001
Present & past within 2 years	446	18%	94	49%	160	29%	116	18%	45	8%	31	6%	
None	1978	82%	98	51%	397	71%	529	82%	492	92%	462	94%	
Missing	982	29%	366	66%	180	24%	147	19%	169	24%	120	20%	
Kaposi’s Sarcoma													0.008
Yes	61	2%	16	3%	17	2%	13	2%	7	1%	8	1%	
No	3345	98%	542	97%	720	98%	779	98%	699	99%	605	99%	

*P-value for trend excluding missing category;

^€^CD4 tests were done in patients who were not ART eligible based on WHO HIV clinical stage

**Fig 1:**
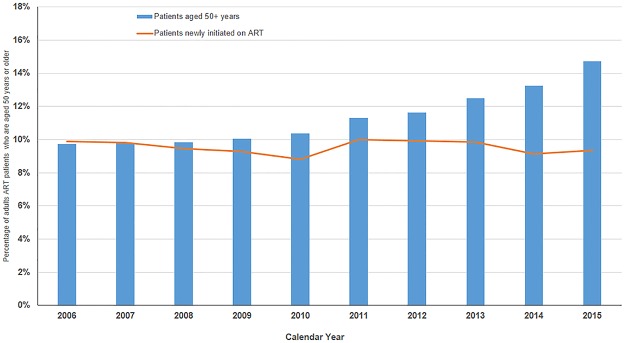
Proportion of older patients (50+years) receiving antiretroviral therapy (ART) at Martin Preuss and Lighthouse clinics between 2006 and 2015.

The proportion of older patients initiating ART care remained stable at 9% while the proportion of active older ART patients increased from 10% in 2006 to 15% in 2015 (Fig 1).

### Immunological response

**Fig 2:**
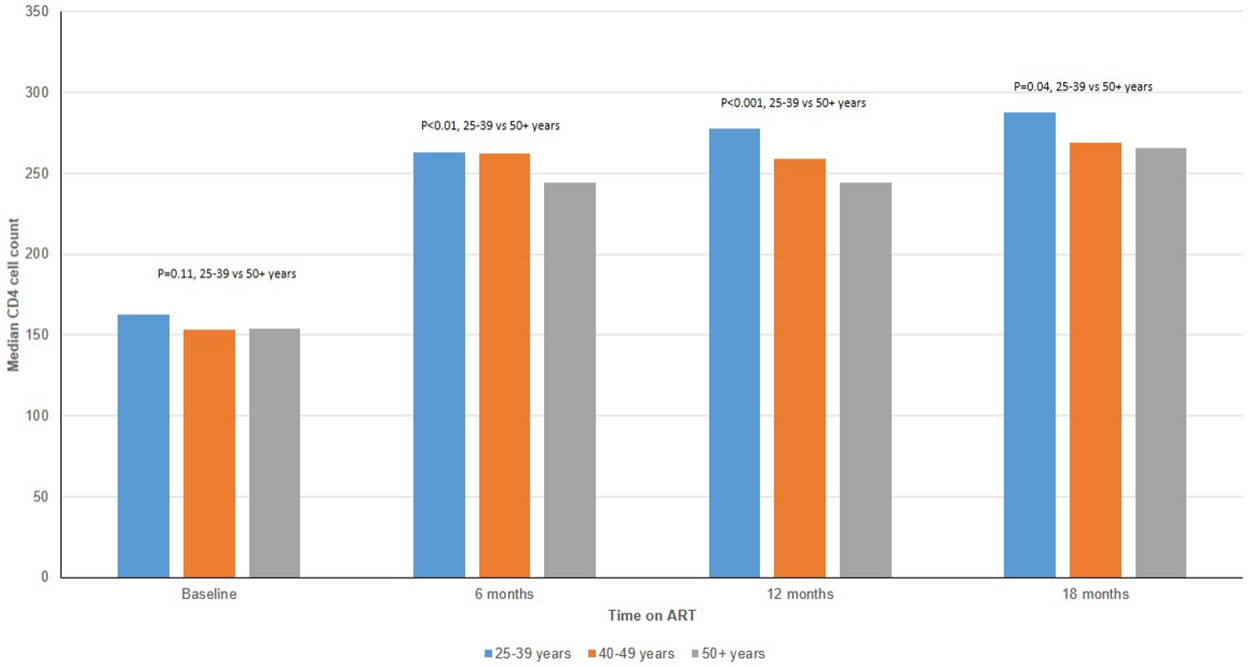
Median CD4 count by age at antiretroviral therapy (ART) and duration on ART.

Given that the Malawi ART program stopped CD4 monitoring in 2013, analysis of CD4 count response was done in ART patients who had ARV visits between Jan, 2006 and Dec, 2012 only. Among those included, CD4 counts were available for 17% (4176 of 23,583), 15% (3012 of 20,039) and 6% (1550 of 19,822) at 6, 12 and 24 months after ART initiation, respectively. Proportion of missing CD4 counts were similar in all age groups. The respective median CD4 cell counts at ART initiation among patients aged 25–39, 40–49 and 50 years and older were 163 cells/μl (IQR 81–244), 153 cells/μl (IQR 74–237) and 154 cells/μl (IQR 80–242), not significant statistically (p = 0.13). However, CD4 cell count response was significantly different over time on ART between older and younger patients (Fig 2).

### Mortality and loss to follow-up

Higher proportion of patients aged 50 years or older (12%) and patients aged 40–49 years (9%) died during follow-up compared to patients aged 25–39 years (9%). Overall mortality rates were 2.3 (95% confidence Interval (CI) 2.2–2.4), 2.9 (95% CI 2.7–3.2) and 4.6 (95% CI 4.2–5.1) per 100 person-years in patients age 25–39 years, 40–49 years and 50 years and older, respectively. At six months on ART, the cumulative probabilities of death were 5%, 6% and 9% while at 24 months, the probabilities of death were 7%, 10% and 14%, among increasing age cohorts, respectively (log-rank test p <0.001).

For mortality comparisons, we compared the factors influencing mortality rates between the youngest and the older age group only. Being male, WHO clinical stage 3 or 4 and BMI <16 were associated with increased risk for death for both the older (≥50 years) and younger patients (25–39 years) in univariable analysis (Table 3). Presence of KS at ART initiation was significantly associated with higher risk of death only in younger patients. In the multivariable analysis, being male, WHO clinical stage 3 or 4, and BMI < 16 were associated with increased risk for death in both older and younger ART patients. Patients aged 25–39 years diagnosed with TB at ART initiation had reduced risk for mortality compared to patients who had no TB at ART initiation while no statistical differences were observed among those aged 50 years and above. KS at ART initiation was associated with higher risk for death in younger patients only.

**Table 3:** Univariable and multivariable model of characteristics associated with mortality among adult ART patients aged 25–49 years and those aged ≥ 50 years.

	Univariable analysis				Multivariable analysis			
Characteristics at ART initiation	25–39 years		≥50 years		25–39 years		≥50 years^Ŧ^	
	RR (95% CI)	P-value	RR (95% CI)	P-value	RR (95% CI)	P-value	RR (95% CI)	P-value
Sex		<0.001		<0.001		<0.001		<0.001
Female	1.00		1.00		1.00		1.00	
Male	2.13 (1.93–2.34)		1.56 (1.27–1.93)		1.61 (1.43–1.81)		1.34(1.07–1.68)	
WHO HIV clinical stage		<0.001		<0.001		<0.001		<0.001
1 or 2	1.00		1.00		1.00		1.00	
3	2.69 (2.35–3.09)		2.20 (1.70–2.85)		2.99 (2.53–3.55)		1.86 (1.41–2.44)	
4	6.47 (5.57–7.50)		3.46 (2.54–4.71)		5.04 (4.15–6.14)		2.61 (1.87–3.64)	
Tuberculosis		0.725		0.073		<0.001		0.189
TB at initiation	1.06 (0.92–1.23)		1.34 (1.02–1.77)		0.57 (0.48–0.66)		1.00 (0.74–1.36)	
TB in the past 2 year	1.02 (0.79–1.32)		0.80 (0.48–1.35)		0.68 (0.52–0.89)		0.62 (0.36–1.08)	
None	1.00		1.00		1.00		1.00	
Kaposi’s Sarcoma		<0.001		0.296		<0.001		0.515
Yes	4.20 (3.60–4.89)		1.38 (0.78–2.45)		1.65 (1.32–2.07)		0.81 (0.42–1.55)	
No	1.00		1.00		1.00		1.00	
Body mass index		<0.001		<0.001		<0.001		<0.001
< 16	3.05 (2.73–3.40)		2.35 (1.86–2.98)		2.38 (2.10–2.70)		2.09 (1.64–2.66)	
16–18.4	1.00		1.00		1.00		1.00	
≥18.4	0.58 (0.48–0.70)		0.60 (0.44–0.82)		0.80 (0.64–0.99)		0.70 (0.50–0.96)	

ART: antiretroviral therapy; CI: confidence interval;

P-value for likelihood ratio test;

^Ŧ^Adjusted for sex, WHO HIV clinical stage and body mass index

Overall LTFU rates were 6.3 (95% CI 6.1–6.5), 4.5 (95% CI 4.2–4.7), and 5.6 (95% CI 5.1–6.1) per 100 person years among increasing age cohorts. The cumulative probabilities of LTFU were 7%, 5% and 7% during the first 6 months on ART, and 18%, 13% and 15% at 24 months on ART among increasing age cohorts (log-rank test p <0.001).

## Discussion

This is one of the first empirical studies from Malawi showing an increase in the proportion of patients aged ≥ 50 years receiving ART and initiating ART with less compromised immune systems over time. Older patients aged ≥ 50 years had slower immunological response to ART in the first 18 months on ART compared to patients aged 25–39 years. Older patients aged ≥ 50 years had double the mortality of younger patients aged 25–39 years. LTFU was lower among those aged ≥ 50 years when compared to younger patients aged 25–39 years.

Similar to previously published studies[2,3], older HIV-infected patients presented with a higher proportion of advanced WHO clinical stages at ART initiation. For those with baseline CD4 counts available for analysis and with WHO stages 1 or 2, the median CD4 cell count at initiation was lower among patients in the older age group although it was not statistically different. Our findings contradict previous Malawian research suggesting that older patients were not sicker than their younger counterparts[16]. Older people living with HIV may access health services less than younger patients [22,23]. As a result, they may be enrolled in HIV care at a more advanced HIV disease stages than younger people. Additionally, healthcare providers may have a lower index of suspicion of HIV infection in older people[24]; therefore, they may be less likely offer HIV tests. Although older people generally tended to present with more advanced HIV disease, we observed a decreasing trend in the proportion of those with advanced WHO clinical stages at ART initiation during the study period. While in 2006 and 2007, 85% of these older patients had WHO clinical stage 3 or 4 conditions at ART initiation, this declined to 41% by 2014 and 2015. Older patients also initiated ART with increasingly higher CD4 counts in later years, therefore, with less compromised immune systems. It is possible that some decline in advanced HIV conditions at ART initiation was due to changes in ART guidelines rather than changes in behavior. Initiating ART with higher CD4 count and/or less immunocompromised can reduce patient‘s viral load to undetectable levels more swiftly than among patients who initiate treatment late [25]. Healthcare providers should intensify provider-initiated HIV testing and counseling among older people and actively link them to HIV care is warranted.

Patients who were 50 years and older showed a significantly lower immunological response to ART in the first 6 to 18 months compared to patients aged 25–39 years. These findings are in concordance with previously published evidence that older patients mount poorer CD4 cell count response than younger patients[13–14]. There was no significant difference in immunological responses between patients aged 25–39 and those aged 40–49. Independent of HIV infection, age is associated with a reduced production of naive T cells and diminished T cell functionality[26].

Mortality on ART among the older patients was twice as high as that among younger patients aged 25–39 years. It is important to note that more patients aged ≥ 50 years started ART with advanced HIV disease. Deaths among these older patients could be related to possible AIDS-related conditions. Similar to another study[13], patients with advanced HIV conditions and male patients had increased risk for mortality in both older and younger age group. While in younger patients, KS was associated with higher mortality, this was not seen in the older group. The reduced risk of mortality in patients diagnosed with TB at ART initiation might be due missed diagnosis at baseline assessment. Patients may be classified as non TB cases but subsequently developed TB when they receive ART hence increasing the risk of mortality among non TB cases.

In industrialized countries, the use of ART has resulted in markedly improved life expectancy in HIV-infected people and a demographic shift in the age compositions of the HIV epidemic towards older ages [27]. Our study provides early evidence of a similar shift in Malawi‘s HIV program. The proportion of HIV-infected people aged 50 years and older receiving ART increased with each successive year between 2006 and 2015. This increase is because of HIV-infected people living longer as opposed to the increased numbers of older HIV-infected people starting ART- as the proportion of those initiating ART at 50 years or above remained stable during the study period. While ageing of the patient population living with HIV is an encouraging indication of effectiveness of ART treatment, it requires a shift in the focus of HIV care. To provide quality care for an ageing population, HIV-related care will need to move away from the traditional management of HIV-related opportunistic infections to increasingly address possible long-term ART-related problems (organ toxicities and longstanding activation of inflammatory cascades with vascular effects), drug-drug interactions and age-related non-communicable diseases(NCDs)[28–29]. A growing prevalence of NCDs complicates HIV care, requiring administration of multiple drugs (e.g. anti-hypertensive and anti-diabetics) in addition to ARVs. The increased pill burden with its effect on adherence as well as potential drug-drug interaction[30,31] may eventually lead to virological rebound. A holistic approach to HIV care involving integration of NCD and consideration of the impact of long-term ART use may begin to address the complexities of caring for aging patients on ART.

While our results are derived from a large, well-documented ART cohort and likely generalizable, the findings need to be interpreted within the context of several limitations. First, CD4 counts were not available for all patients at specific monitoring milestones due to the unavailability of CD4 count testing services. Second, we were unable to report HIV-related mortality as deaths were from all causes. Moreover, we might have underestimated mortality as some deaths may have been classified as LTFU simply because the true status could not be established through tracing. Lastly, we could not adjust mortality and immunological response for non-AIDS diseases as data was not available. Such diseases may be the consequences of poor response to ART.

In conclusion, older patients had advanced HIV conditions at ART initiation, slower immunological responses, lower rates of LTFU, and double the mortality of younger patients aged 25–39 years. Essential enhancement of HIV detection in this age group is necessary to ensure early ART initiation. Given trends in increasing proportion of older PLHIV, there is a need to focus ART care on ageing assocciated diseases including NCDs and degenerative diseases.

## Supporting information

S1 Table Baseline characteristics of adult patients starting ART, by year of ART initiation. (DOCX)
