# Supplementary material for: Characteristics and outcomes of older HIV-infected patients receiving antiretroviral therapy in Malawi: A retrospective observation cohort study
Source: PLoS One. 2017 Jul 7;12(7):e0180232. doi: 10.1371/journal.pone.0180232 (PMC5501463; doi:10.1371/journal.pone.0180232)
Supplement: S1 Table — (DOCX) [file pone.0180232.s001.docx]

| **Characteristics** | | **Year of ART initiation** | | | | | | | | | |
| --- | --- | --- | --- | --- | --- | --- | --- | --- | --- | --- | --- |
| **Age group** | **WHO HIV clinical stage** | **2006-2007** | | **2008-2009** | | **2010-2011** | | **2012-2013** | | **2014-2015** | |
|  |  |  |  |  |  |  |  |  |  |  |  |
| 25-39 | 1 or 2 | 710 | 18% | 1892 | 34% | 2449 | 39% | 2488 | 47% | 2916 | 61% |
|  | 3 or 4 | 3177 | 82% | 3688 | 66% | 3819 | 61% | 2752 | 53% | 1871 | 39% |
|  |  |  |  |  |  |  |  |  |  |  |  |
| 40-49 | 1 or 2 | 209 | 16% | 485 | 29% | 559 | 33% | 670 | 43% | 856 | 58% |
|  | 3 or 4 | 1117 | 84% | 1160 | 71% | 1155 | 67% | 874 | 57% | 614 | 42% |

**S1 Table. Baseline characteristics of adult patients starting ART, by year of ART initiation**
